# Screening for asthma in Cantonese-speaking immigrant children

**DOI:** 10.1186/1471-2458-5-48

**Published:** 2005-05-17

**Authors:** Robyn O Greenfield, Angela C Lee, Roland Tang, Doug Brugge

**Affiliations:** 1 Department of Public Health and Family Medicine, Tufts University School of Medicine; Boston, USA; 2 University College of Citizenship and Public Service, Tufts University, Medford, USA; 3 Department of Pediatrics, South Cove Community Health Center, Boston, USA; 4 Department of Public Health and Family Medicine, Tufts University School of Medicine, Boston, USA

## Abstract

**Background:**

Asthma prevalence among Chinese immigrant children is poorly understood and attempts to screen these children have produced varied outcomes. We sought to learn how to improve screening for asthma in Chinese immigrant children.

**Methods:**

Children (n = 152) were administered the Brief Pediatric Asthma Screen in either Cantonese or English, they then viewed and reacted to a video showing people wheezing and subsequently took a pulmonary function test.

**Results:**

The diagnosed asthma prevalence for our study population was 27.0%, with another 5.3% having possible undiagnosed asthma. Very few children had spirometry findings below normal. In multivariate analysis, being native born (p = 0.002) and having a family history of asthma (p = 0.003) were statistically associated with diagnosis of asthma. After viewing the video, 35.6% of respondents indicated that the images differed from their conception of wheezing. Of four translations of the word "wheeze" no single word was chosen by a majority.

**Conclusion:**

Our findings suggest that asthma diagnoses are higher for Chinese children who were born in the US suggesting that desegregation of data might reveal at risk subpopulations. Care needs to be taken when diagnosing asthma for Cantonese speakers because of the centrality of the word wheeze and the challenges of translation.

## Background

Asthma continues to be a key challenge in the field of pediatric health. With nine million children diagnosed with asthma in the United States, it is the leading cause of school absences, emergency room visits and hospitalizations among American youth [1,2]. Studies of asthma prevalence however, have tended to neglect the Asian-American population, leaving the burden of asthma faced by this population largely unknown [3,4].

Epidemiological studies of asthma have tended to utilize written questionnaires in English or Spanish, focusing on symptoms [1,5]. In the few studies that have included Asian-American children, lower prevalence of diagnosed asthma than in comparable populations has usually been found [1,3,4]. When included as part of "other races" in the Center for Disease Control report, Asian-American children, were found to have an asthma prevalence of 11.4% [1]. Such results come into conflict with results obtained using other, non-written methods. For example a study published in 2002 found 47.1% of Asian-American third-graders in New Jersey to have abnormal spirometry [6].

Recent studies suggest that lack of general asthma knowledge as well as a misunderstanding of the word "wheeze" may affect detection of asthma in Asian children [3,7]. A study of parents of asthmatic children in Nanjing, China found 54.7% of parents had "poor" knowledge of the disease. Parents were unfamiliar with the triggers of asthmatic attacks, the pathology of wheezing and proper management of the disease in general [7]. In a large international study comparing written questionnaire responses and reaction to a video demonstrating wheezing [5], Cantonese speakers showed less correlation between the written and video questionnaire then did English or Spanish speaking subjects, with the inclusion of the video increasing the number of positive responses for Cantonese speakers.

The "gold standard" in asthma diagnosis consists of history, physical examination and spirometry. As the history portion relies heavily on questions utilizing the word "wheeze" and other symptoms of asthma, language could interfere with proper diagnosis.

The word "wheeze" has multiple Cantonese translations, each conveying a slightly different meaning. To our knowledge, previous studies of asthma among Chinese speakers have not reported which translation(s) they used. The translations that we have encountered are: 1)  which means a sound from the throat, 2) () which means a sound from difficulty breathing, 3)  which means a special sound from asthma and is the more professional expression which is used in medical books, and 4)  which literally means a gasping sound made after crying.

A five-question instrument, the Brief Pediatric Asthma Screen (BPAS) has been validated as an additional means of screening for asthma. The BPAS also relies heavily on the use of the word "wheeze," thereby being subject to the same limitation [8]. Two previous studies have used Chinese translations of the BPAS in attempts to determine the prevalence of asthma in Asian-American immigrant children, yielding conflicting results [3,4]. Both studies offered the survey in English and Chinese. In the first study [4], conducted in 2002, parents of kindergarten through fifth grade students completed a self-administered written version of the BPAS. Using, , the professional term for wheezing, this study found the prevalence of diagnosed and possibly undiagnosed asthma to be 16% and 3%, respectively.

In the second study, the survey was administered orally, with surveyors physically demonstrating what they believed to be wheezing [3]. It should be noted that the demonstration more resembled labored breathing and therefore was not specific for asthma. As such, the prevalence of diagnosed asthma in the second study remained statistically the same at 15%, however the prevalence of possible undiagnosed asthma increased over six-fold to 18.6%.

This study seeks to improve the methodology for asthma screening in Chinese-American immigrant children. By use of the BPAS, a video demonstration of wheezing, and spirometry; the study compares the use of current methods to detect asthma in this population. As there were approximately one-half million hospitalizations for asthma in the US in 1995, effective detection of asthma is critical in patient care [9].

## Methods

### Sample

A convenience sample of 152 children ages 5–18, was recruited in the waiting room of the pediatrics department at South Cove Community Health Center in Boston, Massachusetts between June 16-July 23, 2004. Children with cough or fever, who were outside the specified age range, who did not speak either English or Cantonese or who had previously taken the screen were excluded from the study.

### Procedures

The Tufts-New England Medical Center Institutional Review Board as well as the South Cove Community Health Center Board of Directors approved the study protocol. Consent was given orally at time of entry into the study from parents/grandparents/guardians of children less than 11 years of age and the child him/herself for years thereafter. Collected data was anonymous and de-identified. Families were provided with a written description of the study in their choice of English or Cantonese. The protocol was expanded mid-way through the study to include additional data collection. Data from both the "basic" and "expanded" protocol were analyzed and are presented in this paper.

### Questionnaire

A written questionnaire was administered to all participants in the study as our primary tool for assessing asthma status. It was administered to the parent/grandparent/guardian if the child was younger than 11 years of age. Children ages 11–18, were asked to complete the questionnaire him/herself. The survey was written in English and translated into Chinese by one translator using traditional characters. A second translator then translated it back into English in order to check for accuracy. Respondents had the option of taking the survey in their choice of English or Cantonese. A bilingual English/Cantonese speaker (author ACL) orally administered the questionnaire and answered questions when prompted by the participant. The questionnaire consisted of 19 questions.

The majority of questions were demographic in nature. Questions were asked regarding the child's age, sex, place of birth, and length of residence in the United States. Preferred language was inferred from choice of Chinese or English surveys. For children under 11, this meant that it was the parent's preferred language, whereas for children over 11, it was the child's preferred language.

Information was collected on risk factors for asthma, including family smoking habits, highest level of education for parents (as a measure of socioeconomic status), allergies, asthma medication use and family history of asthma. The expanded questionnaire included questions relating to other medical conditions and non-asthma medication use. The remaining five questions in both the basic and expanded questionnaire were from the Brief Pediatric Asthma Screen (BPAS) [8] and were as follows:

1. Have you/your child ever been diagnosed *by a doctor *as having asthma?

2. Have you/your child ever had episodes of wheezing (whistling in the chest) in the *last 12 months*?

3. In the *last 12 months*, have you had/heard your child wheeze or cough during or after active play?

4. Other than a cold, in the *last 12 months*, have you/your child had a dry cough at night?

5. In the *last 12 months*, have you/your child been to a doctor, an emergency room or a hospital for wheezing?

This study used the translation  for wheeze. Though this version literally means a gasping sound made after crying, it was chosen by a bilingual speaker with extensive experience in the Boston Chinatown community to represent wheezing when used in the context of asthma.

### Categorization of asthmatic status

All completed questionnaires were categorized for asthmatic status as described in Wolf et al [8]. An affirmative answer to the first question was automatically categorized as "diagnosed asthma". An affirmative answer to the last question or two or more of questions 2–4, was categorized as "possible undiagnosed asthma." All other responses were considered "probably not asthmatic." For analysis of association between asthma and demographic factors, persons categorized as "possible undiagnosed asthma" were grouped with "probably not asthmatic" to create the non-asthmatic group. The BPAS was administered prior to the respondent's viewing of the video and thus, children were categorized based on the respondent's prior understanding of the word "wheeze."

### Video

We asked all parents/grandparents/guardians or children to watch the "international" version (AVQ 3.0) of the International Studies of Asthma and Allergies in Childhood (ISAAC) Phase One study [5]. Our goal was to provide participants with a visual representation of wheezing on the assumption that they might not understand the concept of wheezing. This brief, one minute, video consists of the following five sequences:

1. "A young person seated with clearly audible wheezing, but without breathlessness and no evidence of airway obstruction

2. Exercise-induced wheezing

3. Waking at night with wheezing

4. Nocturnal coughing

5. Severe attack of asthma"

Respondents were asked if their previous understanding of wheezing on the BPAS corresponded to what they had viewed in the video. An answer of "yes" or "no" was recorded, as were any qualitative responses in regards to the concept of wheezing. Patients screened with the expanded protocol were then asked to re-answer questions 2–5 of the BPAS, basing their answers on the depiction of wheezing in the video. These patients were than categorized again based on their second set of answers to the BPAS. Detailed analyses of the second BPAS scoring are not presented. Cantonese speaking participants taking the expanded protocol were then asked which of the four Cantonese translations best matched the video's depiction of wheezing.

### Spirometry

Pulmonary function testing was performed on all participants willing to attempt it using the ndd Model 2000 *EasyOne*™ Frontline Spirometer (ndd Medical Technologies, Andover, MA), an instrument whose performance correlates well with office-based spirometry [10]. We sought to use spirometry as an objective measure of asthma status. Standard, calibrated scales and stadiometers were used to determine height and weight. Each child was then categorized as underweight (<5^th ^percentile), healthy weight (5–85^th ^percentile), at risk for overweight (85^th^-95^th ^percentile) or overweight (>95^th ^percentile) using the CDC BMI growth charts for boys and girls ages 2–20. Categorization was performed twice, to ensure accuracy.

Spirometry was performed using standard procedures by trained technicians. The children's noses were sealed manually or by use of pediatric size spirometry nose clips. For analysis, we used only spirometry reported by the *EasyOne*™ software as having at least 2 acceptable maneuvers and FEV_1 _readings within 150 ml and FVC within 150 ml (score of "A" or "B"; *EasyOne*™, manual, undated). The parameters recorded were: percent predicted forced expiratory volume during the first second (FEV1), forced vital capacity (FVC), and FEV1 to FVC ratio (FEV1/FVC). Percent predicted values are based upon the results of the NHANES III study as described in Hankinson *et. al*. without adjusting for ethnicity [11].

Criteria for diagnosis of obstructive lung disease were:

FEV_1 _< 80% predicted and FVC < 80% predicted

FEV_1_/FVC < 75%

Numerical values correspond to those recommended by the National Asthma Education and Prevention Program of the National Institutes of Health: National Heart, Lung and Blood Institute [12]. Results of the spirometry were given to the patient and his/her physician so that they could be incorporated into his/her pediatric appointment.

### Data management & analysis

Data was double entered into SPSS version 11.5 and crosschecked for errors by reference to the original hard copies of the surveys and data forms. Chi Square tests were used to generate odds ratios (OR), to demonstrate the likelihood of being diagnosed as asthmatic versus being non-asthmatic for the following variables: birthplace, preferred language of respondent, paternal education, maternal education, smoking in the home, overweight status, allergies and family history of asthma. Due to small numbers of values below 80%, Fisher's exact test was used to examine the relationship of being diagnosed with asthma versus being non-asthmatic with FEV_1 _and FVC values. We then performed a forward step-wise binomial logistic regression to create a model predicting the likelihood of being diagnosed with asthma based on the following independent variables: preferred language of respondent, foreign born, paternal and maternal education, sex, age, country of origin, smoking in the home, allergy, family history of asthma and overweight as possible predictors of diagnosis of asthma. We again used Chi Square tests to elicit odds ratios for having an understanding of wheezing consistent with the video's representation versus inconsistent for the following independent variables: Birthplace, preferred language of respondent, asthmatic status, paternal and maternal education. We performed a forward step-wise binary regression to create a model predictive of consistent or inconsistent understanding of the word wheeze with that portrayed in the video using the following variables: preferred language of respondent, sex, age, overweight, country of origin, foreign born, smoking in the home, allergy, family history of asthma and diagnosis of asthma. For those participants of the expanded protocol we used Mc Nemar's test to examine pre-video BPAS scores with post-video BPAS scores. Statistical significance was set at the p = 0.05 level and borderline significance was set at p ≤ 0.10 for all tests.

## Results

### Participant demographics

Table 1 presents the demographic characteristics of the full study population (n = 152) as well as three subsets, those children completing the basic protocol (n = 63), those completing the expanded protocol (n = 89) and those with acceptable spirometry results (n = 67). Based on BPAS scores, diagnosed asthma prevalence for the full sample was 27.0% with another 5.3% having possible undiagnosed asthma. Children completing the basic and expanded protocols were similar in most ways, however the expanded protocol subset had statistically higher prevalence of family history of asthma (23.9% vs. 11.1%) and lower prevalence of paternal (50.0% vs. 77.1%) and maternal (52.1% vs. 74.5%) education at high school level or above. The subset of children that completed acceptable spirometry did not differ from the total study population demographically.

**Table 1:** Demographic characteristics of the study population and subsets of the population used in the analyses in this study.

	**Basic Protocol (n = 63)**	**Expanded Protocol (n = 89)**	**Total (n = 152)**	**Spirometry (n= 67)**
**Age**				
Less than 11	39.7% (25)	37.1% (33)	38.2% (58)	40.3% (27)
11 and older	60.3% (38)	62.9% (56)	61.8% (94)	59.7% (40)

**Sex**				
Female	50.8% (32)	50.6% (45)	50.7% (77)	58.2% (39)
Male	49.2% (31)	49.4% (44)	49.3% (75)	41.8% (28)

**Birthplace**				
Native-born	57.1% (36)	50.6% (45)	53.3% (81)	59.7% (40)
Foreign-born	42.9% (27)	49.4% (44)	46.7% (71)	40.3% (27)

**Preferred Language**				
Cantonese	61.9% (39)	53.9% (48)	57.2% (87)	55.2% (37)
English	38.1% (24)	46.1% (41)	42.8% (65)	44.8% (30)

**Smoking**				
Smoker in the home	38.1% (24)	35.2% (31)	36.4% (55)	33.3% (22)
Smoke-free home	61.9% (39)	64.8% (57)	63.6% (96)	66.7% (44)

**Allergies**				
Yes	37.1% (23)	37.5% (33)	37.3% (56)	37.9% (25)
No	61.9% (39)	62.5% (55)	62.7% (94)	62.1% (41)

**Family Asthma**				
Yes	11.1% (7)*	23.9% (21)*	18.5% (28)	19.4% (13)
No	88.9% (56)	76.1% (67)	81.5% (123)	80.6% (54)

**Paternal Education**				
Did not complete high school.	22.9% (11)*	50.0% (34)*	38.8% (45)	39.0% (23)
Completed high school or above	77.1% (37)	50.0% (34)	61.2% (71)	61.0% (36)

**Maternal Education**				
Did not complete high school	25.5% (13)*	47.8% (33)*	38.3% (46)	41.4% (24)
Completed high school or above	74.5% (38)	52.1% (36)	61.7% (74)	58.6% (34)

**BPAS Score**				
Diagnosed asthma	22.2% (14)	30.3% (27)	27.0% (41)	26.9% (18)
Possible undiagnosed asthma	7.9% (5)	3.4% (3)	5.3% (8)	3.0% (2)
Probably not asthmatic	69.8% (44)	66.3% (59)	67.8% (103)	70.1% (47)

* p <.05

### Descriptive analysis

Table 2 lists frequencies, ORs and p-values for associations between key characteristics of the study population along with prevalence of diagnosis of asthma. Being foreign born as compared to native born was associated statistically with a lower prevalence of diagnosed asthma (11.3% vs. 40.7%, p = 0.001). Reporting a family history of asthma was associated with a higher prevalence of asthma (53.6% vs. 20.3%, p = 0.001). Borderline statistical associations with diagnosis of asthma were found for lower maternal education, which was associated with lower asthma prevalence (17.4% vs. 31.1%, p = 0.09) and the presence of allergies in the child, which was associated with higher prevalence of asthma (35.7% vs. 21.3%, p = 0.05). There was no statistical association for preferred language of respondent (a variable that combines the parent/grandparent/guardian for children less than 11 and the child him/herself for those over 11 years of age), paternal education, smoking in the home, body mass index, or both FEV_1 _and FVC below 80% of predicted. We could not test associations between asthma diagnosis and abnormal FEV1/FVC ratio because there were only 3 FEV1/FVC ratios below 75%. Associations between FEV1 and sex, foreign born, preferred language of respondent, smoking in the home, allergies, family history of asthma, BPAS score and paternal and maternal education were not statistically significant (not shown).

**Table 2:** Comparison of prevalence of diagnosed asthmatics and non-asthmatics for the total sample (n = 152) by demographic categories.

	**Diagnosed Asthmatics (n = 41)**	**Non-asthmatics (n = 111)**	**OR & 95% CI**	**P value**
**Birthplace **				
Native-born	40.7% (33)	59.3% (48)	5.41 (2.29 – 12.80)	**<0.001**
Foreign-born	11.3% (8)	88.7% (63)		

**Preferred Language**				
Cantonese	24.1% (21)	75.9% (66)	0.72 (0.35 – 1.47)	0.36
English	30.8% (20)	69.2% (45)		

**Paternal Education **				
Completed high school or above	25.4% (18)	74.6% (53)	0.75 (0.33 – 1.72)	0.66
Did not complete high school.	31.1% (14)	68.9% (31)		

**Maternal Education **				
Completed high school or above	31.1% (23)	68.9% (51)	2.14 (0.86 – 5.31)	0.09
Did not complete high school	17.4% (8)	82.6% (38)		

**Smoking**				
Smoker in the home	27.3% (15)	72.7% (40)	1.01 (0.48 – 2.13)	0.98
Smoke-free home	27.1% (26)	72.9% (70)		

**Body Mass Index (BMI) **				
≥ 85 percentile	34.1% (15)	65.9% (29)	1.61 (0.75 – 3.51)	0.22
<85 percentile	24.2% (24)	75.8% (75)		

**Allergies**				
Allergic	35.7% (20)	64.3% (36)	2.06 (0.98 – 4.29)	0.05
Non-allergic	21.3% (20)	78.7% (74)		

**Family Asthma**				
Asthma	53.6% (15)	46.4% (13)	4.52 (1.91 – 10.72)	**<0.001**
No asthma	20.3% (25)	79.7% (98)		

**FEV_1 _and FVC**				
>80%	25.8% (16)	74.2% (46)	0.89 (0.27 – 2.95)	1.00**
≤80%	40.0% (2)	60.0% (3)		

* Odds ratios were computed using Chi-Square tests and are reflective of the likelihood of being diagnosed with asthma for each of the stated demographic variables.

**Fisher's exact test.

### Multivariate analysis

We considered preferred language of respondent, foreign born, paternal and maternal education, sex, age, country of origin, smoking in the home, allergy, family history of asthma and overweight as possible predictors of diagnosis of asthma in a forward stepwise binary logistic regression. Only being native born and having a family history of asthma were statistically significant in the model (p = 0.002 and p = 0.003 respectively), both being associated with higher prevalence of asthma. Because of substantial missing data on parental and maternal education, we also ran the regression leaving these variables out of the analysis. Doing so did not qualitatively change the result (not shown).

### Video screening

Participants were asked to indicate whether or not the video portrayal of wheezing was different from their prior understanding (Table 3). About thirty-six percent (35.6%) indicated that the video was different from their conception of wheeze. Cantonese speakers were more likely to report an inconsistency than were English speakers (47.6% vs. 20.0%, p = 0.001). In a forward stepwise binary logistic regression that included preferred language of respondent, sex, age, overweight, country of origin, foreign born, smoking in the home, allergy, family history of asthma and diagnosis of asthma, Cantonese speakers and overweight children were more likely to say that the video portrayal of wheezing was different from their prior understanding (p < 0.001 and p = 0.045 respectively). For those completing the expanded protocol, post-video BPAS scores were compared to pre-video BPAS scores using Mc Nemar's test, the results were not statistically significant.

**Table 3:** Consistency of understanding wheezing between the video and questionnaire for the total sample (n = 149; 3 missing).

	**Consistent**	**Inconsistent**	OR & 95% CI*	**P value**
**Total**	64.4% (96)	35.6% (53)		

**Birthplace**				
Native-born	68.4% (54)	31.6% (25)	1.44 (0.73 – 2.82)	0.288
Foreign-born	60.0% (42)	40.0% (28)		

**Preferred Language**				
Cantonese	52.4% (44)	47.6% (40)	0.28 (0.13 – 0.58)	**<0.001**
English	80.0% (52)	20.0% (13)		

**Asthma Diagnosis**				
Asthmatic	60.0% (24)	40.0% (16)	0.77 (0.37 – 1.63)	0.494
Non-asthmatic	66.1% (72)	33.9% (37)		

**Paternal Education **				
Did not complete high school.	58.1% (25)	41.9% (18)	1.33 (0.61 – 2.88)	0.756
Completed high school or above	64.8% (46)	35.2% (25)		

**Maternal Education **				
Did not complete high school	54.5% (24)	45.5% (20)	1.54 (0.72 – 3.30)	0.130
Completed high school or above	64.9% (48)	35.1% (26)		

*Odds ratios were computed using Chi-Square tests and are reflective of the likelihood of having an understanding of the word wheeze consistent or inconsistent with the video's depiction for each of the stated variables.

### Translation of "wheeze"

**Figure 1:**
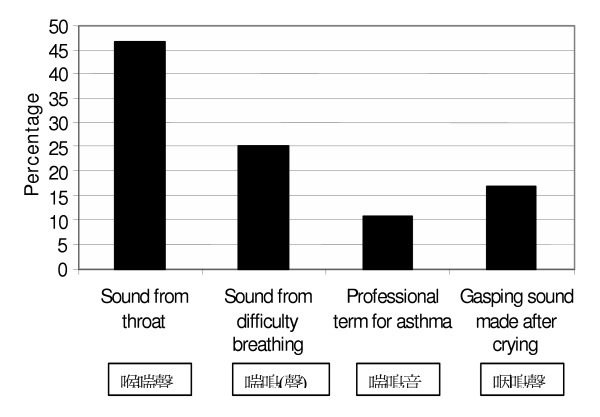
Preferred translation of "wheeze" for respondents in the expanded protocol (n = 47).

The forty-seven (47) Cantonese-speaking respondents completing the expanded protocol rated four choices of Cantonese translations of the English word "wheeze" (Figure 1). "Sound from the throat" was preferred by 46.8%, "sound from difficulty breathing" was preferred by 25.5%, the "professional term for asthma" was preferred by 10.6% and "gasping sound made after crying" was preferred by 17% of respondents.

## Discussion

### Limitations

There are several limitations to our study. We did not ask about smoking by the children themselves, but limited tests of exhaled CO (not shown) suggested that at least a couple smoked. Our measure for socio-economic status (SES) was imperfect, as we had a high non-response rate for the questions of paternal and maternal education level. In most instances this was due to inability of children to recall their parents' education level, but interpretation of educational level may also be limited by the fact that educational systems vary between the US and China. Our classification of preferred language by the parent's choice rather than the child's for children under 11 did not necessarily reflect the preferred language of the child.

Our choice of Cantonese wording for "wheeze" turned out to be one of the less popular options, despite our having introduced it during the BPAS screenings. It is possible that if we had used the most popular choice that it would have improved our BPAS screening. In addition, qualitative reaction of respondents was that the asthma events depicted in the video were more extreme than what the respondents thought of as wheeze. We would suggest that the video might be best described as depictions of "asthma attacks" rather than solely wheezing. Inclusion of spirometry was not an effective tool for assessing asthma status. Possibly the addition of a bronchodilator would have improved its utility.

Our results are not necessarily generalizable because we had a convenience sample from a health clinic waiting room and screened only families that spoke English or Cantonese.

### Interpretation

The population of Chinese-American children that we enrolled in the study reported high prevalence of diagnosed asthma, but appeared to be clinically stable at the time of the screening. By screening out children who were coughing on the day of the screening, we may have underestimated overall asthma prevalence and prevalence of active asthma at screening. Few of their spirometry results were below 75% for FEV_1_/FVC and few were below 80% for FEV_1 _and FVC. It is worth noting that abnormal spirometry is relatively uncommon, even among children diagnosed with asthma [13].

Only 41.5% of the children reported to have diagnosed with asthma would have been categorized as asthmatic by the other four questions in the BPAS screening. We cannot distinguish between the possibilities that there could be misdiagnosis of asthma as compared to prevalent mild or well-controlled asthma. For those children with diagnosed asthma, who would have been picked up by the BPAS, only 7.3% reported use of asthma medication on the day of their screening. Because we did not distinguish intermittent from persistent asthma and we asked only about medication use on the day of screening, we cannot state with certainty what percentage are not receiving optimal care.

We found that asthmatic children were more likely to have allergies and more likely to have a family history of asthma. Both associations are well supported in the literature [14] suggesting that our study population was similar in this way to other populations.

Our strongest finding was that being born in the US was highly predictive of having diagnosed asthma, which suggests that with the Asian-American immigrant population there may be a more vulnerable subpopulation visible only when data is disaggregated. We were unable to determine whether this is a function of true asthma prevalence or differential diagnosis. The BPAS screen showed only 5.3% "possible undiagnosed asthma" cases, suggesting that there is little undiagnosed asthma consistent with the study of Lee et al. [4]. However, difficulty in translating of the word "wheeze" into Cantonese and its centrality in the BPAS leaves ample room for uncertainty about the true prevalence of undiagnosed asthma.

When screening Cantonese-speaking children for asthma we first would suggest using the translation of wheeze that literally means "a sound from the throat". However, we would urge caregivers to also try other translations, as there was not a single clear choice among our study population. Second, and related to the first, we would suggest that the concept of wheezing is not well-understood or popularized among Cantonese-speakers. This, combined with a low level of knowledge about asthma in general, [7] means that the provider needs to be careful when taking a medical history and rely less on the obvious symptom, wheezing. Even for providers treating diagnosed asthmatic children from Cantonese speaking families, we would suggest extensive follow-up to ensure proper understanding and compliance.

### Future studies

There is a need to conduct asthma studies in the Chinese-American population that are free of the limitations of our data collection. This would include asking about children's smoking behavior, use of a better surrogate for socio-economic status (possibly parental occupation, which might be more easily recalled by children than education level), use of the more popular term for wheeze ("sound from the throat") in written or oral screening, and use of a video demonstration with less severe depictions of wheezing.

Additionally, studies are needed to confirm and explain the dramatically different prevalence of asthma between foreign and US born Chinese-Americans that we found. Potential hypotheses for the difference include: differential diagnosis due to language and cultural differences and differences in environmental exposures. Interestingly, exposure to Hepatitis A, a virus more common in China, has been suggested to be protective against the future development of asthma via interaction with the TIM1 gene [15]. If this turns out to be the case, it would suggest a biological cause could explain at least part of the difference in asthma prevalence.

## Conclusion

We hope that this paper raises interest in and prompts examination of asthma among Chinese-American populations. There is a prevailing assumption that asthma is not a problem in this population. If nothing else, our study should bring the validity of such an assumption under scrutiny and raise awareness of the lack of general asthma knowledge in this community. In future studies examining prevalence among Chinese-Americans it is important to consider that prevalence among US born Chinese-Americans appears to be significantly higher than prevalence among foreign-born Chinese-Americans. Failure to desegregate data along these lines may mask the high prevalence of asthma within the sub-group. For both research and clinical practice purposes, the emphasis of cultural understanding needs to be explored. Because of asthma's commonplace stature within American culture at this time, it is easy to take one's level of understanding for granted, thereby missing a potentially life-threatening condition. While we raise this concern with respect to the Chinese-American population, it worth considering that it may hold true for other population groups as well.

## Competing interests

The author(s) declare that they have no competing interests.

## Authors' contributions

ROG participated in the design of the study, helped supervise the fieldwork, conducted data management, conducted the statistical analysis and took the lead in writing the manuscript and assisted in editing it. ACL conducted the field data collection, participated in data management, participated in the analysis and assisted with writing of the manuscript. RT assisted with design of the study, helped supervise the fieldwork, contributed to interpretation of findings and assisted with writing and editing the manuscript. DB provided overall direction to the study, supervised data collection, management and analysis, and participated in writing and editing the manuscript.

## Pre-publication history

The pre-publication history for this paper can be accessed here:


